# Which Are the Most Determinant Psychological Factors in Olympic Shooting Performance? A Self-Perspective from Elite Shooters

**DOI:** 10.3390/ijerph18094637

**Published:** 2021-04-27

**Authors:** Francisco Moreira da Silva, Paulo Malico Sousa, Valter Bruno Pinheiro, Olga López-Torres, Ignacio Refoyo Roman, Daniel Mon-López

**Affiliations:** 1 Sport Sciences Department, Instituto Superior de Ciências Educativas (ISCE), 2620-379 Ramada, Portugal; franciscosilva36@gmail.com (F.M.d.S.); direccaoddesporto@isce.pt (P.M.S.); valter_pinheiro@hotmail.com (V.B.P.); 2 Centro de Investigação em Qualidade de Vida (CIEQV), 2040-413 Rio Maior, Portugal; 3 ImFINE Research Group, Health and Human Performance Department, Facultad de Ciencias de la Actividad Física y del Deporte-INEF, Universidad Politécnica de Madrid, 28040 Madrid, Spain; olga.lopez@upm.es; 4 Facultad de Ciencias de la Actividad Física y del Deporte (INEF—Sports Department), Universidad Politecnica de Madrid, 28040 Madrid, Spain

**Keywords:** rifle, pistol, anxiety, coach, injury

## Abstract

Psychological aspects like anxiety, injuries’ effects, professional psychological support (PPS), psychological training (PT), or athlete-coach relationships could influence shooters’ performance. This study tried to determine which aspects were critical from the shooters’ perspective. Eight elite shooters were interviewed. After using qualitative methods, the following categories were obtained: importance of PT; anxiety and competition relationship; mental preparation; PPS; stress during training; injuries’ psychological effects and coach’s influence. PT is important for shooter’s performance, being PPS a key aspect. Moreover, anxiety levels are critical, raising during the pre-competitive period and oscillating during competition. Furthermore, shooters considered more effective the stress placed on training by the coach than by themselves. Accordingly, the coach plays a key role. Surprisingly, injuries did not affect shooters psychologically, nor in their competitive performance. We conclude that the combination of PPS, shooter competitive experience and the optimal coach’s work can promote a greater performance in Olympic shooting.

## 1. Introduction

Shooting sport requires precise, firm and coordinated actions of many body organs [1]. Angular errors of 0.016° in air rifle and 0.066° in air pistol can lead to not obtaining a score of 10. In main sport shooting events, such as the Olympic Games, World Cups or World and European Championships, a difference of 1.1 points per 60 shots can lead to being a finalist or not [2]. Consequently, a perfect technique of different elements such as grip stability, postural balance, trigger control and aiming accuracy [3] and the ability to handle stress, pressure and anxiety seem to be decisive. This aspect makes shooting a very psychological sport, that demands levels of precision and perseverance close to perfection to win medals internationally [4]. Hence, mental training could be essential due to the attention, control, and precision need in shooting sport.

Many authors point out the importance of psychological training (PT) as a factor that enhances the athletes’ performance. Thus, many studies, report significant performance improvement after mental training [5,6]. PT provides tools to manage anxiety [6] and improving training and competition recovery [7]. In this line, meditation trainings could reduce heart and oxygen consumption rates, which would improve the control of tension and anxiety levels [5]. 

However, if the PT could be crucial in the athletes’ performance, why are there still coaches, athletes and sports managers who do not use the PT? Some studies have pointed out that factors like the lack of knowledge [6], little understanding about PT, lack of time [6,8,9] or the difficult to prove benefits in relation to real performance [10] could be some of the reasons.

The relationship between anxiety and performance have been deeply studied. Shooting is considered a sport sensitive to tension and anxiety [5] and requires extreme mental concentration and precision of movement for success [7]. Accordingly, in situations of high pressure and anxiety, it might not be easy to perform precisely [8]. 

Winning a competition depends, among other factors, on how an athlete interprets and controls his anxiety levels [11,12]. Pre-competitive anxiety level may vary depending on certain factors, such as type of sport, experience, sex and age [12]. Individual sports athletes showed higher levels of anxiety than team sports athletes do due to the individual assumption of responsibility in the results. Furthermore, experienced athletes have lower levels of cognitive and somatic anxiety than less experienced ones. Experienced athletes increased anxiety in the pre-event period and decreased anxiety just before and during competition while less experienced athletes constantly increase anxiety [11]. Nevertheless, there seems to be an ideal level of anxiety for each athlete. Lower or higher levels than optimal ones can impair performance, according to a curvilinear form (inverted U theory) [13]. The Individual Zones of Optimum Functioning Theory proposes that the optimal level of arousal is not always at the midpoint of the arousal continuum [14] and it is individual-dependent. Shooting is more sensitive to somatic changes and fine motor control is required, unlike other sports that involve gross motor skills. That might be the reason why optimal anxiety could be more determinant. On the other hand, a negative relationship between excessive self-confidence and performance has been described. Therefore, high confidence levels can lead to negligence actions and affect performance [15]. Hence, it seems relevant to know the optimal level of anxiety of each athlete individually.

Pre-competitive and competitive situations can elevate nervousness and anxiety even in experienced shooters. In this sense, the PT importance in the athletic performance in relation to the pre-competitive and competitive mental preparation, is widely recognized [16]. Regular PT practice is associated with more successful and consistent performances, especially at the highest levels. Olympic athletes reported that they could have performed better before in their sports if they had strengthened their mental abilities early in their careers [15].

The mental preparation can be divided into cognitive and somatic techniques [17,18]. In this line, some athletes use pre-competitive routines like listening to music, talking to team members or being quieter [19]. These behaviors could be favorable to relaxation/motivation and can help the mind to stay away from negative thoughts [13]. 

Consequently, in addition to the necessity of technical and physical trainings [20], athletes, coaches and sports psychologists should use cognitive or somatic coping strategies to reduce pre-competitive state anxiety, maintain and focus concentration, regulate arousal levels, increase confidence, and maintain motivation and peak performance according to their modalities [6,11,21].

Based on the previous evidence, professional psychological support (PPS) would seem relevant to help athletes in the efficient use of PT and to improve performance. The sport psychologist can teach psychological skills and help athletes to improve motor skills, deal with competitive pressures, adjust the level of awareness and stay focused among the many distractions of the competitive environment [8] reducing anxiety and athletes’ negative feelings [16]. Moreover, the sport psychologist must know the different sport situations, the psychological resources of the athletes and technical team and evaluate their needs in order to optimize performance [19]. For these reasons, PT should be an integral part of an athlete’s holistic training process, along with other training elements [8]. Multidisciplinary teams are common in other sports, with the presence of coaches, physiotherapist, doctors, psychologists, kinesiologists, among other professionals but this is not common in elite teams in Olympic shooting. 

During competition, the difficulty of coping under pressure can decrease the skills control, leading to a poor performance [22], so could stress during training be useful to train stress situations during competition? It seems that training in a high stress environment is an effective method to cope with anxiety and improve competition [22,23]. 

Interestingly, sensory stimuli of voluntary movements during training should replicate the environmental cues of competition to reach an ideal learning [24]. Consequently, simulating performance actions under pressure could increase competence and individual’s context-specific confidence [22]. In addition, athletes who considered that they have resources and effectiveness to deal with pressure conditions perceived anxiety as a performance facilitator [21,22]. As anxiety can be consider both, a facilitator or an added stress factor, to apply PT techniques that make anxiety a facilitator, could be interesting for coaches and shooters.

All these stress situations can increase the risk of suffering an injury. Although Olympic shooting has a low injury rate (one of the sports with the lowest risk of injury during the London 2012 Olympic Games), they can interfere with performance at some level. Besides the physical limitations, injuries’ pain can restrict the athlete’s ability to focus on performance, preventing to compete in optimal conditions [25]. On the other hand, pain can be perceived as routine or benign, and normalized [26]. One way or the other, when it happens, can also affect shooters’ mental health [27]. Thus, shooters may experience depression, anxiety, lack of self-esteem, poor feeling of recovery, lack of self-confidence, competitive insecurity, distrust about their own level of expertise and fear of failure [1]. This mental status could also affect the rehabilitation process [26]. The application of mental strategies during rehabilitation has been showed very effective [28]. For these reasons, coaches and physiotherapists seem to play an integral role in psychological recovery from injuries [26]. Nevertheless, and despite the scientific evidence, it is not clear if it is regularly included, together with physiotherapists in injuries recovery programs. 

Specific literature shows that the coach is one of the most important factors affecting the athlete’s development and progress, but a multidisciplinary team could help performance in some ways. The coach’s ability to improve the athlete’s learning and optimal progress has become one of the main factors of the athlete’s performance [29]. Thus, the coach not only teach and instruct skills, techniques and tactics [30], but is also a leader, psychologist, friend, teacher, people manager, administrator and model [31].

The coaches’ incentives and praises are positively related to the satisfaction, competence, intrinsic motivation, and performance of the athletes. Accordingly, training literature have suggested that the most challenging and beloved coaches are those who demonstrate the knowledge to manage the athletes’ needs and problems, building a positive relationship through trust, friendship, availability and care about athlete´s wellbeing [32], including mental health.

Although some of the mentioned topics have been addressed in previous sport literature, some questions remain unanswered in the specific field of elite shooters, especially from a qualitative point of view: Do shooters really know the advantages of having PPS? Are shooters aware of the most appropriate PT for sport shooting? How does anxiety influence performance and which is the optimal level? How injuries affect performance? Can the coach replace the psychologist? Which are the characteristics to be expected in a beloved coach? Consequently, the main objective of the study was to determine which psychological aspects affect the shooting performance from the shooter perspective themselves.

## 2. Materials and Methods

### 2.1. Participants

Four men and four women elite Olympic shooters aged 42 ± 10.54 years with a competitive starting age of 22.8 ± 5.73 years participated in the present study. The participants belonged to four different countries: Spain, Portugal, Mexico and Brazil. Descriptive characteristics as well as professional trajectories (participants profiles, number of participations in 1st level international competitions, times finalist and medals) are shown in Table 1. All participants were selected via social networks and via personal contacts of the researchers [20]. Two inclusion criteria were used to consider the elite level of the shooters: (A) have at least once participation in the Olympic Games and (B) meet at least one of the following requirements: (B1) have been selected by their country to participate more than 50 times in top-level competitions being a finalist at least one time or (B2) have participated more than 30 times in top-level competitions being a medalist at least one time. In addition, to ensure that the selected shooters met both criteria, their results were checked in the International Sport Shooting Federation official webpage [33]. Shooters who did not met the two inclusion criteria were excluded from the study.

After being informed about the nature and purpose of the study, all participants signed an informed consent. The authors certify that the present study was carried out in the absence of any potential conflict of interest. The study was conducted in accordance with the Declaration of Helsinki, and the protocol was approved by the Ethics Committee of the Polytechnical University of Madrid.

### 2.2. Script of the Interview

A semi-structured script (see Supplementary File S1) was used to minimize the possible bias and to standardize the interviews. All the method process was carried out following the study of Mon-Lopez et al. [20]. The interview script included open questions. The topic of the questions was selected by consensus of two researchers, who were expert shooting coaches with international experience, after a scientific literature review. After the topics were selected, both researchers design together the number and the specific text of the questions. This process was carried out through periodic meetings between the two researchers (approximately once per week). Once the first version of the script was done two external international referees (one expert in qualitative methods and one expert in shooting sport) jointly reviewed the interviews and their feedback were used to modify the script interview. Reliability of the final script was tested twice in a pilot study with two shooters who gives additional feedback to improve the script interview from the athletes’ perspective. Finally, the researchers wrote the final version of the semi-structured script used in the study.

### 2.3. Interviewer and Interview Procedure

One week before the interview, the script was sent to the participants so that they could suggest new questions or ask any question. A single interview per day was scheduled to avoid possible fatigue of the interviewers. Two Olympic shooting experts conducted the eight semi-structured online interviews. All interviews were conducted simultaneously by the same two researchers in order to ensure the same process during the study. Both interviewers were Olympic shooting coaches with wide international experience. This fact improved the use of shooting specific terminology and helped create a good environment between interviewers and shooters. The interview was conducted online by Skype and had a minimum duration of 40 and the maximum of 86 minutes. Interviews were recorded with a WS 852 MP3 recorder (Olympus, Tokyo, Japan) for later transcription.

### 2.4. Categories

Initial areas of knowledge and possible categories were created according to a deductive analysis of the previous literature and the final interview script. However, after interpreting the interviews and the subsequent analysis of the answers related to psychological variables, seven different categories were obtained [34]: (1) Importance of PT, (2) Relationship between anxiety and performance, (3) Pre-competitive and competitive mental preparation, (4) PPS, (5) Stress during training; (6) Psychological effects of injuries and (7) Coach’s influence on the athlete.

### 2.5. Data Analysis

This study used qualitative methods that involved an explorative approach to inquiry [35]. The data analysis consisted in four phases: (I) a full transcription of the interviews, (II) a meticulous selection of the phrases and words, (III) elaboration of each category and (IV) a summary of the answers and frequencies of each idea according to each category. Two interviewers made the transcription, which was revised by other researcher (who acts as a referee) to reach high levels of agreement in case of discrepancy. To analyze de transcriptions and the frequencies of each related idea the Nvivo v.10 software (QSR International, Burlington, MA 01803, USA). was used. Two researchers analyzed the verbal reports separately and selected the categories. Cross-triangulation was performed by the experts (interviewers) and revised by one referee who also solved any discrepancies between the experts. During this process one category was removed (from eight to seven). Once the categories were determined all the concepts, words and phrases were ordered in their respective categories and quantified.

## 3. Results

The results of the present study were divided into seven different categories related to PT in shooting sports. Additionally, two of the eight shooters never practiced any type of PT. However, three out of eight athletes have always been irregular PT practitioners, while three out of eight are regular PT practitioners with professional psychological support.

### 3.1. Importance of PT

All participants, even athlete 1 and 8 who do not practice PT, considered PT very important. All shooters, except athlete 1, mentioned that PT helps reduce, deal with and control anxiety levels. This opinion gains relevance since it is frequently expressed throughout the interview (see Table 2).

*At the beginning it cost me a lot to control my anxiety and after I learned techniques to be able to relax and calm my anxiety … It can help you a lot with the fears you have in competition… That is why I say that PT is very important*.

(Athlete 2)

### 3.2. Relationship Between Anxiety and Performance

#### 3.2.1. Competition Anxiety Levels

For athletes 2, 3, 4, 5 and 7, being a little anxious at the start of a competition is important. These anxiety states, although not measured, keep athletes in an alert state allowing them to be more prepared for competition. This topic seems to be very important due to the number of times that shooters mentioned this concept during the interview (see Table 2):

*It depends on how you manage your emotions and in addition, it can influence you positively or negatively… I am, for example, the opposite. In competition I manage it well and generally I compete at the same level as I train. I even overcome myself competing in relation to training*.

(Athlete 4)

Contrary, athlete 1 declared not to feel anxiety during competitions while athletes 6 and 8 reported negative effects of anxiety on competition, seeing no advantage in being anxious:

*During the competition, I expend my time to technical management and much less time to worry about the results. Soon it seems that the stress will be minimal and the anxiety null*.

(Athlete 1)

#### 3.2.2. Competitive Experience and Its Influence in the Control of Anxiety Levels

The competitive experience, except for shooter 2, is important because helps shooters have a better mental control and reduce their anxiety levels throughout their sports career.

*The competitive experience has helped me to control and even that anxiety disappeared before and during competitions*.

(Athlete 6)

#### 3.2.3. Physiological Changes Related to Anxiety

All shooters reported to have had some physiological changes (i.e., “somatic anxiety”) related to the competition throughout their sports careers, namely: increased heart rate; discomfort (burning/cold/tightness); restlessness, nervousness and agitation; muscle tension, tremors and shortness of breath (see Table 2). However, through competitive experience and/or PT and PPS, they were able to better manage levels of anxiety.

*I feel restlessness. Now, the heart rate only increases when the competition starts, but I remember starting competitions with my knees shaking, shaking all over*.

(Athlete 5)

#### 3.2.4. Variation in Anxiety Levels from the Previous Week, Up to and during the Competition

For athletes 1 and 6, there are no significant variations in anxiety before and during competition.

*Definitely, the competitive experience has helped me to control and even don’t feel anxiety before and during competitions*.

(Athlete 6)

On the contrary, for shooters 2, 3, 5, 7 and 8, there is a progressive variation in anxiety levels until competition. For shooters 2, 3 and 8, anxiety increases especially when the competition goes wrong and for shooters 5 and 7, anxiety can also increase even if the competition is going well:

*The highest point of anxiety was when the competition started. It goes down if the competition goes well and increases if it went bad*.

(Athlete 2)

Shooter 4 tried not to think about the competition during the week and his wish was for the competition to start as soon as possible:

*I just want to compete as soon as possible. Regarding the week before I try not to think about the competition until I am there, and I only worry about preparing everything that I may need during the competition*.

(Athlete 4)

### 3.3. Pre-Competitive and Competitive Mental Preparation

Athletes 2, 3, 4, 6 and 7 reported that in pre-competitive and competitive moments, they use mental preparation techniques such as breathing exercises, visualization and listening to music. Athletes 1, 2, 4 and 7 also tend to be quieter the day before and the day of the competition. Athlete 1 also usually goes to bed earlier and Athlete 7 also likes to watch videos. (See Table 2).

*I would say that is a matter of visualization too. Breathing, I think, is very important to calm down our body a little, always in the pre-competition moment the heart beats harder, that feel of cold in the stomach*.

(Athlete 3)

On the other hand, athlete 5, prefers to talk with team members and athlete 8, concentrates more on competition (see Table 2):

*I prefer to be talking than to listening to music, alone or focused … I like to talk more*.

(Athlete 5)

### 3.4. Professional Psychological Support (PPS)

Regarding PPS, shooters 3, 4 and 6, reported to have psychological support, considering it very important for their performance. This concept seems to be very relevant for all the shooters because the idea of PPS and PT, appeared often in the interview (see Table 2):

*Yes, right now I have a good psychologist*.

(Athlete 6)

*Yes, I have a sports psychologist who helps me today*.

(Athlete 3)

Contrary, shooters 1 and 8 mentioned not having PPS. Shooter 1 justified it due to the lack of knowledge and having no need and shooter 8, due to a lack of knowledge and time:

*From my point of view, I don’t do it for lack of knowledge and need*.

(Athlete 1)

Shooters 2, 5 and 7, reported that the psychological support they had throughout their careers was very little and sporadic and that their federations gave them very little support. Athlete 5 also mentioned that most coaches think that is not important:

*I have had very little psychological support in my entire career… I had PPS in 94, it was cool, and after that, it was practically very sporadic… Whit that work I learned to relax, I learned to breathe, I learned to meditate, this for me is a job that I carry since 94 in my whole life*.

(Athlete 7)

In addition, shooters 2, 5 and 7 stated that if they would have had psychological support, they would have taken less time to achieve their current results:

*I could learn to do it on a one-year journey with a psychologist, perhaps, but it took me 10 years to get there*.

(Athlete 5)

### 3.5. Stress during Training

Regarding this topic, two situations were considered; the stress placed on training by the athlete himself when training alone and the stress on training placed by the coach. Seven out of eight of the interviewees reported that the stress placed by the trainer simulates competition better than training alone (see Table 2). Shooter 4 added that it depends a lot, on what you want to achieve, and on the emphasis you want to place on the shooter’s stress:

*With the coach, it is much easier to reach that situation, however it is very difficult to train alone and to create a stressful situation*.

(Athlete 2)

Athletes 1, 2, 3, and 6 also mentioned that the implementation of stress during training can be greater with the presence of the team or more shooters.

*In training sessions with the whole team, we can do finals and of course I get excited because I always want to win*.

(Athlete 6)

On the other hand, shooters 1 and 4, who normally train alone and without a coach, mentioned that they try to implement some type of stress that simulates competition:

*Close to competitions, I do competition training simulations. Noise around, I put on background music, because now with the new regulation they put on background music. I put music at different volumes to adapt myself, as it is not the same to shoot in total silence or to shoot with music that activates your body a lot*.

(Athlete 4)

### 3.6. Psychological Effects of Injuries

In this regard, during their sports careers, athletes 1,3, 4 and 8 had no injuries, athlete 5 had a minor elbow and shoulder injury, and athletes 2, 6 and 7 had more serious injuries. Athlete 2 had injuries to the elbow and shoulder, athlete 6 had injuries to the shoulder and elbow and later to the cervical spine and shooter 7 had injuries to the shoulder and elbow (see Table 2). None of the four injured athletes, with more or less severity, were affected psychologically or impaired in their performance while competing injured. In relation to shooter 6, at times of competition, she had greater fatigue, but she was able to overcome physical pain by thinking about a recent family loss:

*The injury made difficult for me to train but did not limit my result due to pain or some physical limitation*.

(Athlete 7)

*Not at a psychological level, because in the shooting position I have no pain, I feel more tiredness, less reaction … But I can do things, as they should be done*.

(Athlete 5)

### 3.7. The Coach’s Influence in the Athlete

All athletes reported that the coach has a great influence on the athlete, both at a technical and at a personal level. Shooters highlighted the influence of the coaches comments, their role as a friend, the importance of praise at the right time, their ability to help overcome negative situations (both in training and in competitions), as well as their ability to maximize the athlete’s performance (see Table 2):

*Yes, when I finish a competition, and I’ve done it well, it is important to me that my coach congratulates me. It is a very important part for me that my coach feels satisfied with my work*.

(Athlete 6)

*When it went wrong while we competed, you could go out to talk to your coach and any word he said to you could stop what you were doing … he would say words to you and break what you were doing and get in again well*.

(Athlete 2)

## 4. Discussion

This study tried to determine the importance of PT in Olympic shooters from their own perspective. Although the sample is small, it is important to remember that participants were top elite shooters who have participated in at least one Olympic Games. In addition, it is important to investigate elite athletes in minority sports where the literature is scarce, especially from the shooter’s perspective, like in the present study, to give coaches important information that can be used to improve athletes’ performance. 

All 8 shooters from the present study considered PT very important in performance, what agrees with the results from [5,9]. They also manifested that PT could help to regulate anxiety levels, deal with pressure, perform a better technique, increase or have a more regular performance and avoid fears, similar to other authors like [1,16,19]. Therefore, our results agree with most of the studies that concluded that PT contributes to optimize sport performance [6,8,16,19].

By developing effective strategies to deal with adverse situations, the athletes are placing themselves in an individual zone of optimal functioning [14], dealing adequately with pressure and improving technically. Consequently, according to Dosil [19], PT helps to develop successful training strategies that allow the athlete to better face competitive pressure. It is important to keep in mind that there is not an absolute optimal anxiety level. Due to individuality, each athlete would have his/her optimal level of anxiety. It should be part of the training to find this level and modulate and adjust it to obtain the best performance. Consequently, PT could help shooters to find this level and adjust it.

Mostly, athletes declared to be able to cope with competition anxiety levels, exceeding their results in relation to training, what is in agreement with other authors [6,14,36]. In our study, five out of eight shooters consider important to have moderate levels of anxiety during competition to activate an alert state. It seems like the optimal arousal and anxiety level do not affect performance but helps improving it. Nevertheless, it might not happen in every athlete. Two shooters of the present study reported anxiety negative effects on competition. Interestingly, previous literature has showed that the 85% of the elite athletes interpret anxiety as facilitative contrary to the 15% debilitating [37]. Additionally, the anxiety effects on performance seems to be related to other aspects like the athlete’s age and gender [38], the sport type [11] or the PPS [8]. Consequently, the effects of anxiety can be positive and facilitating or negative and debilitating, depending on how the shooter interprets the changes [13,14,15] and the amount of anxiety that is optimal for each person [13].

Interestingly, most of the shooters (7/8) agreed that the competitive experience allows them to better cope with anxiety and have better mental control, demonstrating lower patterns of cognitive and somatic anxiety when compared directly with less experienced athletes. In this line, similar results were obtained by [12,39]. In addition, depending on their levels of expertise, more experienced athletes could develop better coping strategies, increasing self-confidence and experiencing anxiety symptoms as competitive facilitators [40]. Together with experience, PPS could help control physiological changes linked to anxiety (increased heart rate, shortness of breath, tightness or burning in the stomach, tremors, muscle tension, nervousness and nausea). These results would be in line with previous studies which indicated that the competitive experience significantly influences the perception and direction of competitive anxiety, as well as the intensity of self-confidence, both for individual and collective sports [16,41].

Our results showed that athletes experience competitive anxiety differently. Most athletes feel an increase in anxiety until the beginning of the competition, varying depending on how the event is unfolding. A possible explanation for these results could be found in the pre-competitive expectations that athletes seem to develop for each competition. In fact, causal attributions in the sport with negative or unexpected results could increase anxiety [39]. Therefore, PPS seem to play a fundamental role, through the psychological training programs adapted to the needs of the athletes, depending on their levels of expertise and experience. Accordingly, the shooters in the present study reported to use breathing exercises, visualization and listening to music. These somatic techniques as pre-competitive routines may play a fundamental role in the athlete’s development [17], anxiety reduction [18] decreasing pre-competitive stress and improve sports performance [7]. 

Regarding PPS, all shooters considered it important. Surprisingly, only (3/8) athletes confirmed having a psychologist. The shooters who did not have PPS and/or do not practice PT, pointed out the following reasons: lack of time, lack of knowledge, negligence of coaches, lack of high-performance structure and lack of support from federations. Hence, these shooters’ reason would agree with the previous literature [6,8,10].

According to Gee [9], one of the reasons for not using PPS seems to be the lack of knowledge about the process and the mechanism of how PT affects performance. However, it has been probed that the use of PPS, improves the athletes’ knowledge, making they recognizes its importance in sports performance and accordingly adding the PPS as part of their microcycles [6].

Regarding the injuries’ effects on performance, our results (50% of the shooters injured anytime) differ in terms of the frequency of injuries with the previous literature, since shooting is a sport with a low injury rates [27]. However, this difference could be due to the period analysed in both studies, as our study considered all sports life while Engebretsen et al. [27] explored one competition period. 

Previous literature showed that sports injuries and the associated pain can have psychological effects on athletes or restrict their ability to focus on performance [25]. Contrary, in our study, 4/8 shooters reported having had an injury, but none reported having been affected psychologically or even altered their performance. In addition, a female shooter was able to overcome her physical pain during the competition, even with a serious injury, associating this pain with a family loose. Consequently, this fact may be associated with a dissociative strategy [26] more used by women [42] and confirming some differences in shooting between men and women [43].

As a rule, the coach plays a key role in the training process. Our results would confirm this topic in Olympic shooting. Thus, the relationship between coach and athlete seems to be based not only on technical and tactical aspects, but also on good interpersonal relationships. Similar results were found already by other authors [30]. Coach must be aware of his behaviour to improve the athlete’s psychological adaptation since his interaction strategies could influence the athlete’s emotions. This fact can be observed in many situations. For example, shooters consider the stress implemented by the coach during training much more effective than by the athlete himself. Specifically, an athlete mentions that training without pressure is neither efficient nor effective. In this sense, according to the previous literature, our results indicated that training with pressure should be used by coaches [23].

Although this study includes a novel self-perspective of eight Olympic shooters, some limitations should be mentioned. Even though several topics have been addressed we do not have enough information to pointed out specific guidelines for PT. Furthermore, Olympic shooting has many modalities and we have only analyzed precision ones. In addition, only a limit number of countries (four) from Europe and America were analyzed, consequently our results must be taken with caution as other countries or continents could have different structures and ways of working. Lastly, although the reliability of the data is high due to the high professional level of the participants (all Olympics), there is not much literature from the shooter’s perspective to compare with. Thus, further researchers could be conducted to complement our data.

## 5. Conclusions

In general, all shooters agreed that PT contributes to the development of sports shooting performance and can regulate anxiety levels, help deal with pressure, perform the technique better and increase or have more regular performance. In addition, the effects of anxiety on competition can be positive or negative depending on how shooters interpret changes during competition. In this line, the competitive experience associated with the mental control would allow the shooter to deal better with anxiety.

Having a PPS was not common among the shooters, although some of them had PPS in the past, what allows them to be able to practice autonomously. Shooters agree that if they had had PPS early in their careers, they would have obtained results more quickly. Accordingly, elite shooters usually do visualization and breathing techniques, listen to music, or are quieter and more focused on the competition in the pre-competitive periods. This absence of PPS is due to the negligence of many coaches and the lack of athletes’ time for dedication and support from Federations and other national entities.

For elite shooters, the coach has a key role in the simulation of stress strategies for competition, being the coach’s pressure superior to the stress implemented by their self when athletes train alone. In addition, the coach must be credible, inspire confidence and promote a healthy relationship with his athletes, otherwise it will harm them. Lastly, the injuries/pain does not appear to have affected psychologically or altered the performance of the injured elite shooters. 

As a practical application, federations and national entities responsible for shooting, should take into account athletes’ psychological support, including it as a training component, to the extent of technical, economic and organizational possibilities, to promote and improve shooters’ performance.

## Figures and Tables

**Table 1:** Participants profiles, number of participations in first level international competitions, times finalist and medals.

Athlete	Sex	JJOO/YOG	WCH	EUCH/AMCH/PAN Games/EU Games	WC	FWC
1	M	5P	6P	35P	52P	7P
		2F	3F	27F	29F	8F
		–	1S	3G, 2S, 8B	4G, 5S, 5B	2S
2	F	5P	4P	22P	52P	5P
		1F	–	3F	27F	5F
		–	–	–	1G, 4S, 4B	1B
3	M	1P, 1P (YOG)	3P	4P	29P	1P
		1F, 1F (YOG)	1F	2F	3F	–
		1S, 1S (YOG)	–	1G, 1S	2G	–
4	M	2P	5P	13P	58P	3P
		–	2F	6F	8F	3F
		–	1G	1G, 1S, 1B	1B	1B
5	F	1P	4P	23P	25P	–
		–	–	1F	5F	–
		–	–	–	–	–
6	F	2P	4P	8P	27P	5P
		1F	–	7F	7F	4F
		–	–	3S	1G, 1S, 3B	2G
7	M	2P	5P	9P	39P	–
		–	3F	22F	5F	–
		–	1G,1S,1B	7G, 7S, 3B	–	–
8	F	1P	7P	25P	19P	–
		–	–	–	1F	–
		–	–	–	–	–

Abbreviations. M = male, F = female, JJOO = Olympic Games, YOG = Youth Olympic Games, WCH = World Championship, EUCH = European Championship, AMCH = American Championship, PAN Games = Pan–American Games, EU Games = European Games; WC = World Cup, FWC = Final World Cup; P = number of participations in the competition, F = times being finalist in the competition. G = gold medals won, S = silver medals won, B = bronze medals won, X = arithmetic mean, SD = standard deviation.

**Table 2:** Concepts and words most repeated by the athletes interviewed in the psychological area.

Category	Ideas/Concepts/Words	Sh 1	Sh 2	Sh 3	Sh 4	Sh 5	Sh 6	Sh 7	Sh 8	Total
**PT**	Importance psychological training	3	8	4	3	7	8	5	8	46
	Improve Results	5	1	3	3	4	1	5	1	23
**RAAP**	Anxiety	8	18	14	4	15	15	8	7	89
	heartrate/heart speeds up/high heart rate	1	1	1		2			1	6
	Nervousness/muscle tension	11	8	1	11	1		16	2	50
	Competitive experience	6	6	1		2	1	4	1	21
**PCMP**	Breath	0	6	1	2	2	1	13		25
	Visualization	0	0	4		7	8	3		22
	Music	3	2	2	7	3	3	1		21
	Read	0	0	0	1	0		0		1
	Psychological training/psychology	10	11	12	13	16	15	13	10	100
	Concentration/Meditation/Mental	27	0	3	7	2	16	5	16	76
**PPS**	Psychological support/psychologist	1	5	4	5	6	11	14		46
**SDT**	Pressure and stress placed by the coach	6	11	4	1	2	2	4	*2*	32
	Sloppy/more relaxation					1		1		2
**PEI**	Injury/Pain	7	3	9	5	1	15	6	4	50
	Difficult/tired//reaction/decrease training/fatigue					5	2	6		13
**CIOA**	Influence/confidence/relationship/calm down	1	1	2	7	3	1	2	1	17
	Reward/praise/congratulate						5			5
	speak/comment/words	1	6	3					2	12
	Coach	4	15	6	8	19	17	10	3	82

Abbreviations. Sh = Shooter. Importance of psychological training (PT), Relationship between anxiety and performance (RAAP), Precompetitive and competitive mental preparation (PCMP), Professional psychological support (PPS), Stress during training (SDT), Psychological effects of injuries (PEI), Coach’s influence on the athlete (CIOA).

## Supplementary Materials

The following are available online at https://www.mdpi.com/article/10.3390/ijerph18094637/s1, File S1: Questions for those who practice psychological training and who do not practice psychological training.
