# Supplementary material for: Which Are the Most Determinant Psychological Factors in Olympic Shooting Performance? A Self-Perspective from Elite Shooters
Source: Int J Environ Res Public Health. 2021 Apr 27;18(9):4637. doi: 10.3390/ijerph18094637 (PMC8123879; doi:10.3390/ijerph18094637)
Supplement: Supplementary file 1 [file ijerph-18-04637-s001.zip › ijerph-1169072-supplementary.pdf]

Questions for those who practice psychological training	Questions for those who do not practice psychological training
1. What is your opinion about the psychological component in sports shooting? How can this psychological component influence the results?<br>2. Do you feel that training under pressure made by the trainer or by the athlete him or herself, is important to train for the competition pressure?<br>3. Do you think that the self-induced pressure by the athlete can have the same effect as that created by the coach (individually and / or in group)?<br>4. Does your training include the psychological component? Who guides this psychological component (the shooter, a psychologist, the trainer, etc.)? It has always been like this?<br>5. What would be the impact on your sports performance if you did not have psychological support?<br>6. In the period before a competition (for example in the previous week), do you use any mental training strategy? Which one?<br>7. To what extent can mental training contribute to dealing with competitive pressure and / or anxiety?<br>8. What reasons have led you to understand the importance of psychological training in sport shooting?<br>9. How can compliments, comforting words or reproaches from your coach affect you during training, competition or after a competition?<br>10. How do you prepare psychologically for a competition between the training and competitive period?<br>11. What emotional reactions do you have between pre-competitive moments and the competition? What strategies do you use to deal with this?<br>12. When you are anxious, what physiological reactions do you feel in your body due to that anxiety?	1. What is your opinion about the psychological component in sports shooting? How can this psychological component influence the results?<br>2. Do you feel that training under pressure made by the trainer or the athlete him or herself, is important to practice for the competition pressure?<br>3. Do you think that the self-induced pressure by the athlete can have the same effect as that created by the coach (individually and / or in groups)?<br>4. Does your training include the psychological component? It has always been like this?<br>5. What would be the impact on your sports performance if you had psychological support?<br>6. In the period before a competition (for example the week before), do you use any type of mental strategy? Which one?<br>7. To what extent can mental training help cope with competitive pressure and / or anxiety?<br>8. How can compliments, comforting words or reproaches from your coach affect you during training, competition or after a competition?<br>9. How do you prepare psychologically for a competition between the training and competitive period?<br>10. What emotional reactions do you have between pre-competitive moments and the competition? What strategies do you use to deal with this?<br>11. When you are anxious, what physiological reactions do you feel in your body due to that anxiety?<br>12. How does your anxiety vary during the week previous a competition?<br>13. Did your competitive experience and background help you to overcome anxiety better?

13. How does your anxiety vary during the week previous a competition?<br>14. Did your competitive experience and background help you to overcome anxiety better?<br>15. What strategies do you use to focus on the day of the competition? Music, reading, meditating, talking to the family, etc.<br>16. In the weekly training, how long do you train the physical, technical, and psychological parts daily, on average? Do you think it is enough? From your own perspective, how many hours/days do you think would be necessary to train each part (physical, technical and psychological)?<br>17. Have you had any muscle injuries since the beginning of your sports career? If so, which one? What was the origin of this injury? Did you have any more injuries?<br>18. Did any injury affect you psychologically?<br>19. In your opinion, do you think that anxiety can have positive effects on the shooter's performance?<br>20. Is there a question that has not been asked and that you would like to have been asked?	14. What strategies do you use to focus on the day of the competition? Music, reading, meditating, talking to the family, etc.<br>15. Have you had any muscle injuries since the beginning of your sports career? If so, which one? What was the origin of this injury? Did you have any more injuries?<br>16. Did any injury affect you psychologically?<br>17. In your opinion, do you think that anxiety can have positive effects on the shooter's performance?<br>18. Are there any questions that has not been asked and that you would like to have been asked?
